# How the study of online collaborative learning can guide teachers and predict students’ performance in a medical course

**DOI:** 10.1186/s12909-018-1126-1

**Published:** 2018-02-06

**Authors:** Mohammed Saqr, Uno Fors, Matti Tedre

**Affiliations:** 1 College of Medicine, Qassim University, Qassim, PO Box: 6655, 51452 Kingdom of Saudi Arabia; 2 Department of Computer and System Sciences (DSV), Stockholm University, Borgarfjordsgatan 12, PO Box 7003, SE-164 07 Kista, Sweden; 3 School of Computing, University of Eastern Finland, PO Box 111, Joensuu, Finland

**Keywords:** Collaborative learning, E-learning, Social network analysis, Computer-supported collaborative learning, Blended learning, Clinical, Case discussions, Learning analytics

## Abstract

**Background:**

Collaborative learning facilitates reflection, diversifies understanding and stimulates skills of critical and higher-order thinking. Although the benefits of collaborative learning have long been recognized, it is still rarely studied by social network analysis (SNA) in medical education, and the relationship of parameters that can be obtained via SNA with students’ performance remains largely unknown. The aim of this work was to assess the potential of SNA for studying online collaborative clinical case discussions in a medical course and to find out which activities correlate with better performance and help predict final grade or explain variance in performance.

**Methods:**

Interaction data were extracted from the learning management system (LMS) forum module of the Surgery course in Qassim University, College of Medicine. The data were analyzed using social network analysis. The analysis included visual as well as a statistical analysis. Correlation with students’ performance was calculated, and automatic linear regression was used to predict students’ performance.

**Results:**

By using social network analysis, we were able to analyze a large number of interactions in online collaborative discussions and gain an overall insight of the course social structure, track the knowledge flow and the interaction patterns, as well as identify the active participants and the prominent discussion moderators. When augmented with calculated network parameters, SNA offered an accurate view of the course network, each user’s position, and level of connectedness. Results from correlation coefficients, linear regression, and logistic regression indicated that a student’s position and role in information relay in online case discussions, combined with the strength of that student’s network (social capital), can be used as predictors of performance in relevant settings.

**Conclusion:**

By using social network analysis, researchers can analyze the social structure of an online course and reveal important information about students’ and teachers’ interactions that can be valuable in guiding teachers, improve students’ engagement, and contribute to learning analytics insights.

**Electronic supplementary material:**

The online version of this article (10.1186/s12909-018-1126-1) contains supplementary material, which is available to authorized users.

## Background

Over the past few decades, the use of technology-enhanced learning (TEL) has become increasingly ubiquitous in the health education sector. TEL has the power to transcend the boundaries of space and time, offering convenience, efficiency, and cost-effectiveness. It also facilitates networked learning by means of computer-supported collaborative learning (CSCL)—features that have been demonstrated to positively enhance learning when coupled with properly designed resources [1].

The benefits of collaborative learning have for long been recognized. It may support learners, diversify understanding among students and educators, and provide a stage for cooperation in a positive atmosphere that fosters the building of learning communities [2]. Well implemented collaborative learning can facilitate knowledge construction, and encourage involvement and motivation of learners in the learning process [3–8].

Social constructivists see humans as social creatures who grow up by developing knowledge and skills through interactions with different communities. They assume that learning is a social byproduct of conversation and negotiation with peers and that learners acquire knowledge by participating in relevant social activities or working collaboratively in groups [9, 10]. Connectivism—a theory developed to address learning in the digital age—asserts that knowledge and learning exist in a multiplicity of viewpoints and that learning as a process occurs by connecting sources of information. It values the role of communication and information appraisal skills in the development of learning and staying up-to-date [8, 11].

When implemented in stimulating learning environments using authentic activities, interaction with other students may help learners make deeper and more meaningful knowledge construction. It becomes particularly effective when learners are encouraged to respond to arguments, negotiate concepts, debate points of views, contribute to ideas, and share insights and alternative perspectives to the discussed topics [3, 4, 12–17].

An online asynchronous discussion board (forum) is a tool for CSCL that offers the opportunity for students to interact and cooperate in online communities [18]. Forums establish a platform for dialogue among peers and educators that facilitates reflection and exchange of ideas. In that way, learners can build on ideas posted by their peers and learn collaboratively [17, 19–21].

Forums have become a standard feature of all the key LMSs. However, the built-in analytics dashboards of most major LMSs offer limited insight into studying interactions among students. Those limited insights are in the form of statistics and frequency of participation. For instance, the Moodle™, and Blackboard™ LMSs report only the number of views and posts by each participant in each forum, whilst lacking the capabilities for studying the patterns of interactions and the structure of communication. Such features might be better analyzed through visualization and social network analysis, which can be obtained only through external applications or plugins [22].

### Social network analysis

Social network analysis (SNA) is a distinct type of analytics that can be used to map relations and interactions between actors within groups in participatory environments [22–26]. Advocates of social network analysis emphasize the role of the social structure, one’s position in the community, and one’s relations and interactions as important factors that shape one’s behavior and performance, in addition to attributes such as age, gender, and disposition [27]. SNA has been used across a wide variety of disciplines. For example, in criminology, SNA has been used to study collaboration between offenders, patterns of criminal behavior, and gang rivalry [22]; in management, SNA has helped assess organizational communication hierarchies, the flow of information, and the decision-making process [22]; and in medicine, SNA has facilitated the study of how infectious diseases propagate [24] and the exploration of connections in human gene networks [28]. In academia, SNA methods are well established in the study of scientific collaboration, co-authorship, and citation analysis [29]. Despite this widespread use in various fields, the use of SNA in education is still limited [23, 25, 30]. The analysis of social networks is commonly performed through two types of methods: network visualization and quantitative analysis.

### Network visualization

A social network is represented by what is commonly known as a *“sociogram”* or a graph. A sociogram is a graphical mapping of relations and interactions between actors in a network. Each actor—a student or a teacher in the learning context—is denoted by a *node* in the graph, and a relationship or interaction between actors is denoted by an *edge* [22]. Sociograms can be directed (where each interaction is mapped from one node to another) or undirected (where there is no certain direction of the interaction) [31].

**Fig. 1:**
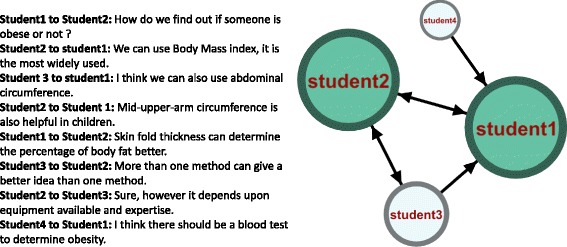
the left side discussion mapped using SNA. Each node (circle) corresponds to a student, each edge (arrow) corresponds to an interaction, the arrowheads represent the direction of the interaction, and the size of circles is proportional to the total number of interactions (degree centrality) and color intensity represents the role a participant connects (comes in-between) others and mediates their interactions (betweenness centrality)

To demonstrate how SNA works, Fig. 1 below shows a graphical mapping (sociogram) of a discussion (a group of interactions) among four students. The graph demonstrates the possibility to sum all interactions and roles in the discussion in one graphical representation (sociogram) by means of SNA.

Using SNA to visualize learning networks, educators can have an easy-to-understand bird eye overview of all discussions and social interactions in a course [32], active and inactive students [32, 33], instructor role and learning design [34], the flow of information, and efficiency of group work [35].

Monitoring online interactions can reveal patterns that are amenable to meaningful intervention. An instructor who uses SNA to monitor a discussion thread can have an outline of interactions and might be able to stimulate an inactive discussion by promoting a collaborative dialogue [36, 37]. Isolated students who are at risk of poor performance can be identified [38, 39], and facilitated through inclusive online environments, well-designed collaboration scripts, improving networking skills, increased social capital, and rewarding collaborative group learning [35, 40–43]. In a similar way, instructor dominated networks can be identified. These networks are characterized by instructor-centered interactions, few student-to-student interactions, and low level of knowledge construction [23, 33]. Intervention might help promote interactions among students and encourage teachers to facilitate rather than dominate. Using SNA can assist in determining those non-participatory patterns and possibly monitor the intervention [23, 25, 43]. Insights generated by SNA in a course can also support course redesign in subsequent iterations [44].

### Network quantitative analysis

Network quantitative analysis is a mathematical method to quantify the connectedness and relations of actors in a network. *Centrality* is the construct used to indicate how prominent a particular node is in a network or how important is that node to the communication of information [37]. Interest in using centrality measures as predictors of student achievement or group performance has arisen with the emergence of learning analytics as a discipline [37, 45]. Romero et al. [46] used in-degree (total number of incoming interactions) and degree centrality (total number of incoming and outgoing interactions) measures to predict students’ performance and found that most students who passed the course had obtained high centrality scores. Hommes et al. [47] found a positive association between degree centrality and student learning; that association was stronger than previous grades, social integration, and academic motivation. Gašević et al. [45] found closeness centrality (how reachable or close a student is to his peers) to positively correlate with higher grades. Ángel et al. [48] found a correlation between centrality measures and performance in some courses and negative in others. They concluded that these mixed results do not suggest that SNA predictors are useless, but rather call for further research to identify the context in which SNA centrality measures might work as reliable predictors. In a trial to assess the role of network position in predicting performance, Joksimović et al. [45] found weighted degree (total number of interactions, taking into account the quality or strength of interactions) centrality to be the most significant factor. Similar to Ángel et al. [48], Joksimović et al. [14] attributed the differences between their findings and those of others to the context in which the study was based.

Peer to peer interactions are important in promoting engagement and enhancing the learning process by enabling the student to establish functional relationships, construct meaning, and understand concepts through discourse and reflection [14, 36]. Increasing interactivity among peers has been reported to promote higher achievement [37]. Thus, the study of social interactions in online collaborative setting may be of potential value in predicting academic performance. Factors such as social capital, prominence, and students’ roles as information-brokers might also add to the available indicators and help obtain more accurate predictive learning analytics in relevant contexts [13, 41, 49].

The quality of collaboration and interactions in a course have been found to affect the whole course learning environment as well as students’ performance [8, 45]. Using visual analytics has the potential to further enhance our understanding of the status and dynamics of online collaborative clinical discussions, ensure that the learning environments are collaborative and engaging for learners, as well as allow us to identify the factors that enhance participatory behavior or situation where intervention is needed [3, 4, 12, 22, 50].

Although social network analysis techniques have been used for a broad range of disciplines and purposes [22], the field’s applications are still rare in education in general, and medical education in particular [5, 23, 51]. The aim of this work is to assess the potential of visual and statistical social network analysis to study clinical case discussions and to evaluate how the course’s social structure and user parameters might help predict student performance.

### The research questions of this study were

1. What information can the study of the social structure provide about the status of online collaborative learning on the course, discussion, and individual levels?
2. Which network parameters correlate best with students’ performance?
3. How can student’s position, interactions, and relations in a network be used to predict his or her final performance?

## Methods

### Context

This study was designed as a case study that applied social network analysis on students’ interactions within a blended medical course. The course was a Surgery Course of the second term 2015 at Qassim University, College of Medicine, Saudi Arabia. This was a term-long clinical course that used clinical case discussion (clinical case scenarios or patient problems) as a teaching strategy to enhance clinical competence and engage students [52]. The discussions used the Moodle learning management system (LMS) forum module as a platform for interaction, and they were moderated by the instructor. The cases were designed to help achieve the objectives and intended learning outcomes of the course, which were: using clinical reasoning to understand the patient’s problem to reach a diagnosis and be able to create an appropriate management plan, as well as improve one’s capability to work efficiently in a team and use information and communication technology for learning and evidence-based practice of medicine.

Data collection and analysis of this study followed the data mining method of Romero et al. [53], which is divided into four steps:

### Data collection

Structured Query Language SQL was used to extract interaction data from the Moodle LMS database and export it to a table (spreadsheet). The data extracted were user ID, forum ID, parent forum, author of the post (source), author of the reply (target), time created, time modified, subject, post content, and student group. Data also included students’ final grades and midterm grades.

### Data preprocessing

The data were cleaned by removing corrupted records (two records were deleted due to missing the target of interaction), participants’ names were anonymized, students were coded as S1 to S35, and data were converted to a format compatible with the SNA application “Gephi” for import [53]. Two datasets were created: the first covered the whole course duration and the second covered the first midterm.

### Data mining and analysis

Data were visualized and analyzed using Gephi 0.91 software. Gephi is an open source SNA application that can be used for network visualization and analysis [53]. It has multiple algorithms for network visualization, of which *Forced Atlas 2* was used. It uses a force directed layout that draws each node based on its relations and connections with other nodes. As such, structurally related nodes are rendered closed to each other. This technique allows a better visualization and interpretation of the overall network structure. Gephi’s main advantage over other applications is its “dynamic mode,” which enables researchers to visualize network evolution and time of events and reflects changes in node position in real-time [54].

### Performance

Students’ final grades were used to measure final course performance, and midterm grades were used to measure performance up to the midterm. Objective Structured Clinical Exam grades were used to measure clinical performance, and multiple-choice questions (MCQs) were used to measure knowledge comprehension, analysis, and application. Students were classified either as underachievers who are at risk of failing (lowest 1/3) or achievers who are relatively safe from failing (top 2/3).

### Statistical analysis

Since network data are known to violate the traditional assumptions of conventional statistics (normal distribution and independence) [55], we chose Kendall’s Tau-b test to measure the correlation coefficient between ranked variables. The test was performed using permutation methods by *PAST* (Paleontological statistics software package for education and data analysis); the permutation test was based on 9999 random replicates. SPSS software version 24 was used to perform automatic linear regression (ALM) and binary logistic regression. ALM offers improvement in key areas, namely better variable selection, handling of extreme values (outliers), as well as merging of similar predictors and conducting ensemble methods [56]. SPSS was also used to perform a stepwise backward binary logistic regression model for the prediction of underachievers.

### Network quantitative analysis

We calculated the parameters that correspond to the size of the network, the extent of the students’ activity in the network, group connectedness, and cohesion. The parameters calculated for each network were:

- *Network size*: the number of nodes.
- *Average degree*: the mean degree centrality of all group members. The average degree is a quantification of the average level of interactivity of participants [44].
- *Network density*: the ratio of actual interactions between peers to the total possible; in contrast to the simple quantification provided by the average degree or the network size, network density is a relative indicator that increases as more members participate, and thus points to the diversity of participation, collaborative behavior, and group cohesiveness [44].
- *Average clustering coefficient*: the average clustering coefficient of the group members; an indicator of the tendency of group members to interact together [44].

### User parameters

Because there are different criteria for node importance or prominence, centrality can be calculated in various ways depending on the context [44]. In this study, we calculated centrality measures that reflect three groups of constructs:

### The quantity of interactions

- *In-degree centrality* is the number of interactions an actor receives, and it is considered an indicator of popularity or prestige. Students who have high in-degree centrality usually have influence or prestige [44].
- *Out-degree centrality* is a measure of outgoing interactions from an actor. It is a quantification of the interactions a student makes and is an indication of how active the student is in the network [44].
- *Degree centrality* is the sum of incoming and outgoing interactions of an actor, and it is calculated by summing out-degree and in-degree centralities [44, 57].

### Role in moderating and relay of information

- *Betweenness centrality* is a measure of the actor’s involvement in moderating interactions; it is measured by counting the times a participant comes “in-between” others. By doing so, the participant connects the unconnected peers and thus facilitates communications and acts as a bridge or broker of information exchange [44, 58].
- *Information centrality* is a measure of the importance of a node in information flow and network cohesion. Information centrality of an actor is defined as the relative drop of network communication efficiency if this actor was removed. A student with high information centrality often has a prominent role in information exchange and communications [59].
- *Closeness centrality* is a measure of how close an actor is to the other collaborators in a network. It is calculated as the inverse of distance between the participant and all other peers in the networks. Close actors can quickly interact with others and are easy to reach [44, 57].

### Connectedness

- *Eigenvector centrality* measures the importance of an actor taking into consideration how well connected the neighbors of the actor are. Connections to well-connected or important actors in the network translate to higher values of Eigenvector centrality [44]. Eigenvector centrality is one of the methods used to estimate the social capital and the influence of one’s ego network.
- *Eccentricity* measures how far an actor is from other actors in the network and can be viewed as an indication of isolation. Students with high eccentricity scores are expected to be less connected to others in the network and difficult to reach [47].
- *Clustering coefficient* measures the overall tendency of a student to work with peers in the group; it is calculated as the proportion of actual edges between a node and its neighbor peers to the total possible edges that can be achieved [26, 44].

### Interpretation and evaluation of the results

The results were analyzed using two different methods:

1. Visualization of students’ interactions and interaction patterns on three levels:

- Course level: to have an overall view of the status of collaborative learning in a course, information-giving, and information-receiving networks, and the role of the teacher and students in the discussions.
- Discussion thread level (discussion thread is a group of interactions under the same topic): to have an outline of interactions in individual discussion threads.
- Learner level (ego networks): to map the social profile of students.

2. Network analysis: extraction and interpretation of network metrics and how they are linked to performance.

## Results

### Research question 1

What information can the study of the social structure provide about the status of online collaborative learning on the course, discussion, and individual level?

The course included 35 students with one instructor (36 nodes). The data were drawn from 34 discussion threads, and the data set totaled 1251 interactions (forum posts). These interactions were visually and mathematically analyzed on course level, discussion thread level, and individual learner levels as follows:

### Visualization

#### At the course level

Interpretation of social networks depends on the context and the design of the course where it occurs [33], and the interactions can be mapped in different ways. One approach is to visualize the interactions to represent participants’ roles in terms of quantity and influence in order to provide a general idea about the course structure and participants’ roles.

**Fig. 2:**
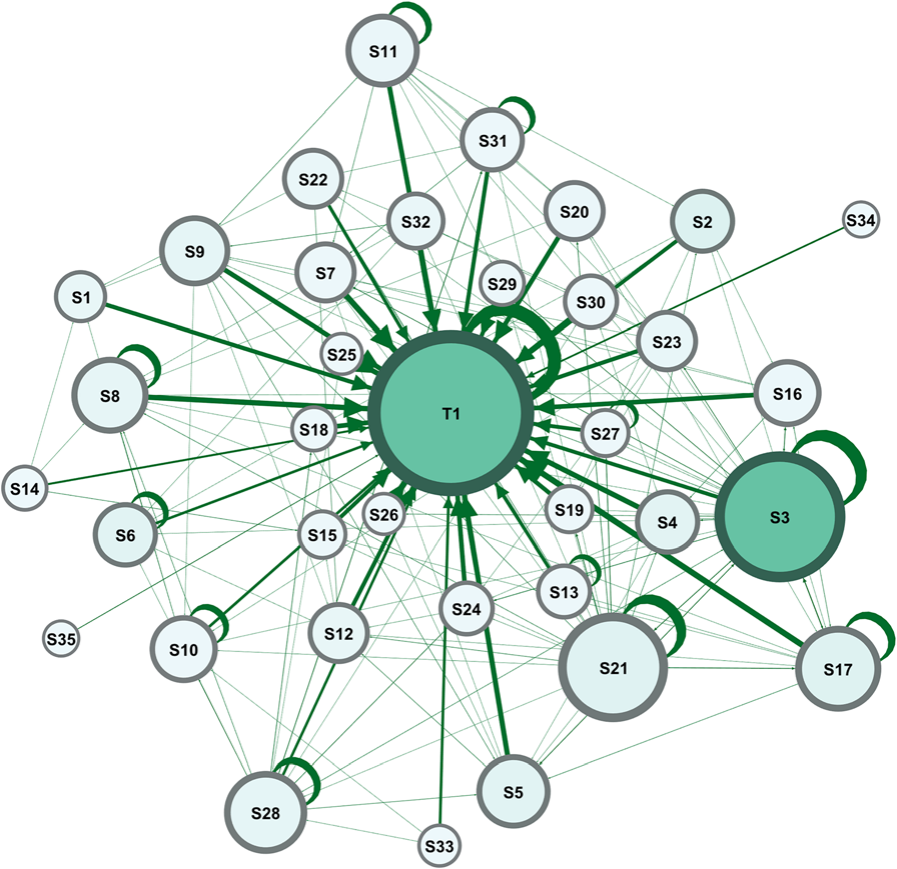
a graph that summarizes all interactions in the course. The figure shows the instructor (T1) being central to all interactions and receiving most connections (highest prestige). Each node (circle) corresponds to a participant, each edge (arrow) corresponds to an interaction, the arrowheads represent the direction of the interaction, the size of each node is relative to its degree centrality, color intensity represents betweenness centrality, and the thickness of edges represents the frequency of interactions

The interactions were mapped on four different sociographs. First, an overall course network summed up all posts in a single graph, which outlined the structure of the course and the patterns of interactions. Second, an information-giving graph showed how information spread from students to the instructor. Third, an information-receiving graph highlighted the nodes’ levels of receiving interactions. Fourth, a centrality graph was plotted using the information centrality parameter of each participant to demonstrate roles of participants in the flow of information. A time-lapse video was created to visualize the evolution of network over the whole duration of the course. The first graph, shown in Fig. 2, demonstrates the overall course network.

The graph in Fig. 2 shows the instructor (represented by the node T1) with most edges pointing to him, which indicates that he is receiving most interactions (high in-degree). Actors who receive most interactions act as leaders in a network (highest prestige or authority). The instructor also has the largest node size (highest degree), and his node is the darkest (highest betweenness centrality). This pattern where the instructor is the most influential and the target of most interactions is recognized as instructor-centered. Since the design of this course relies on interactions usually started by the instructor, it was intended that students reply to the instructor when trying to solve the clinical cases, so the graph is well aligned with the instructional design of this course.

The presence of interconnections among students is a sign of a considerable amount of debate and interactions among students trying to establish their cognitive and social presence [14, 16]. It is also apparent that some students (e.g., S3 and S21) have high degree centrality represented by larger node sizes (an indication of higher activity). However, S3 has a more influential role with high betweenness centrality (dark color), and S3 is important to the flow of information across the network. There are two outliers (S34 and S35) with small node size and few connections, which indicate low activity and isolation.

**Fig. 3:**
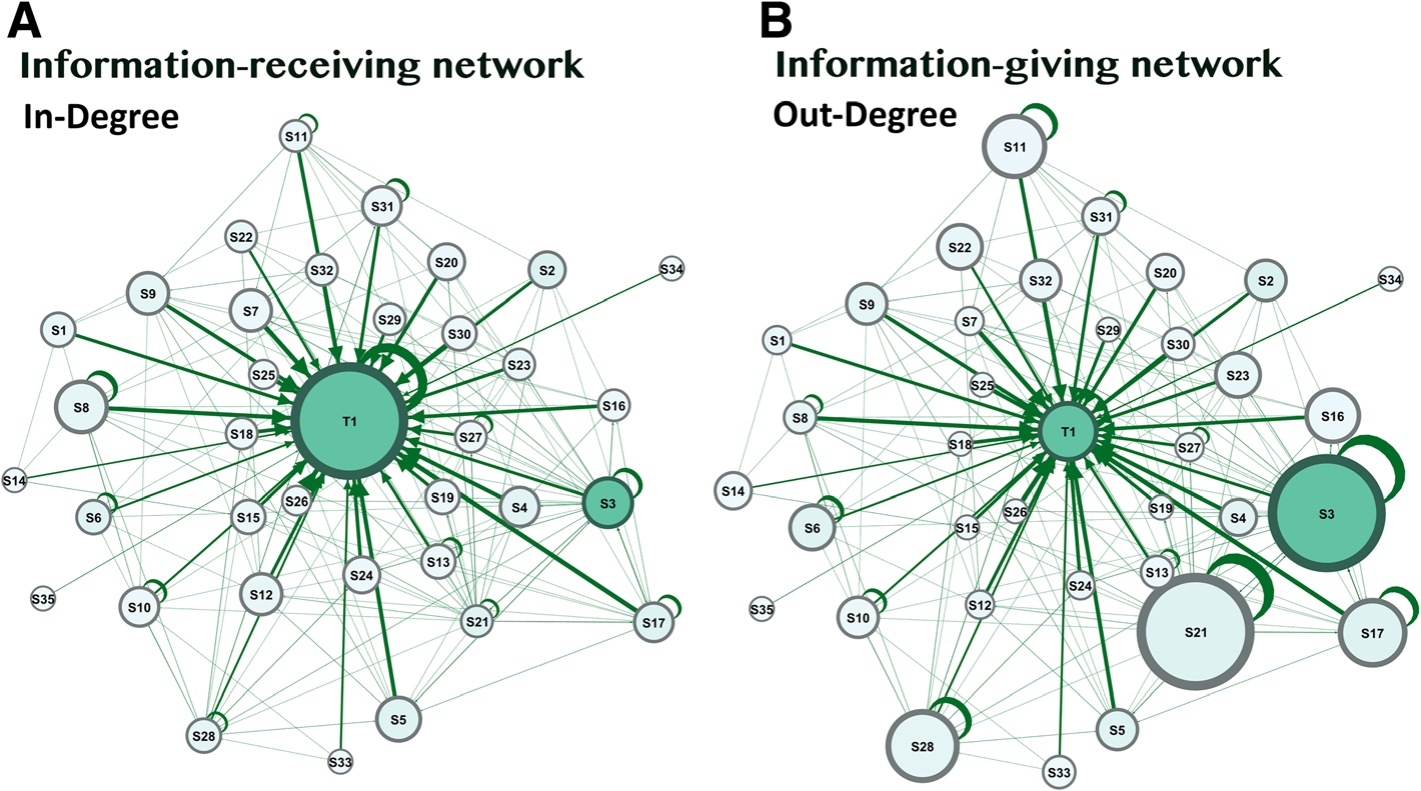
Information giving versus information receiving network. To the left, in (**a**) node size was configured by in-degree centrality (received interactions) to demonstrate the information receiving network, where students who received more interactions will have larger nodes. In (**b**) node size was configured by out-degree centrality (outgoing interactions) to demonstrate the information giving network, where students with more outgoing interactions will have larger nodes

Figure 3a demonstrates the *information receiving network*, where node size was configured by in-degree centrality (received interactions). The figure shows that the instructor received more interactions than any student did (highest prestige). A few students received interactions, but they remained with a much lower prestige than the instructor. In Fig. 3b, node size was configured by out-degree centrality (outgoing interactions) to demonstrate the *information giving network,* where students with more participation have larger nodes. The figure shows that most students were actively participating in discussions, and the network was dominated by students like S3, S21, S11, S28, and S17, who had the highest *prestige*, and whose prominence was superior to that of the instructor. When compared side by side as in Fig. 3, the information-giving network is more collaborative, it shows more active students, and it shows a moderate role for the instructor. The information-receiving network shows an instructor dominating the discussion over all the participants.

**Fig. 4:**
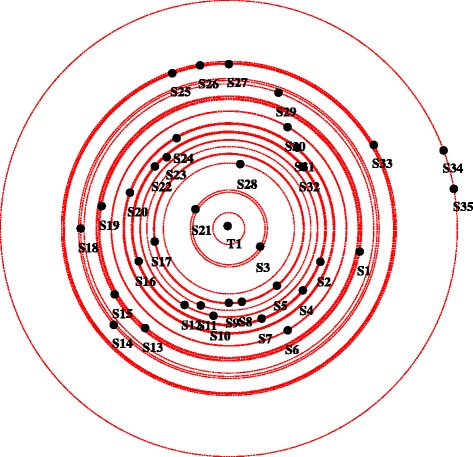
Information centrality graph. Each circle represent a node, circles near to the center of the plot have higher information centrality and thus are more influential in information transfer

To view how information is transferred and who are the principal brokers, an information centrality graph was plotted. In Fig. 4 the instructor, S3 and S21 are closer to the center because they had a central role in brokering information.

Dynamic network changes over time can give insights about the course interactions at various points of time. Additional file 1: Video S1 shows how the community forms during the first week (network formation stage). From the second week until around the mid-course, students are actively engaged and responding to the instructor (engagement stage). The engagement slows down just before the mid-term exam for a brief period of time, but after the exam, the engagement resumes, and this time the network interactions are more mature than before, as more interactions are occurring among students. During the final week, students are expectedly disengaged. The video also clearly shows individual students’ interactions in real-time throughout the course.

#### At discussion thread level

Similar to visualizing interactions at the course level, visualizing individual discussion threads reveals information about the principal actors and patterns in individual discussion threads. The importance of profiling an individual discussion is that it is the building unit of the course network. Diagnosing gaps or pitfalls in dysfunctional online group collaboration starts at the discussion thread level, and if an intervention would take place, it usually happens at a discussion level.

**Fig. 5:**
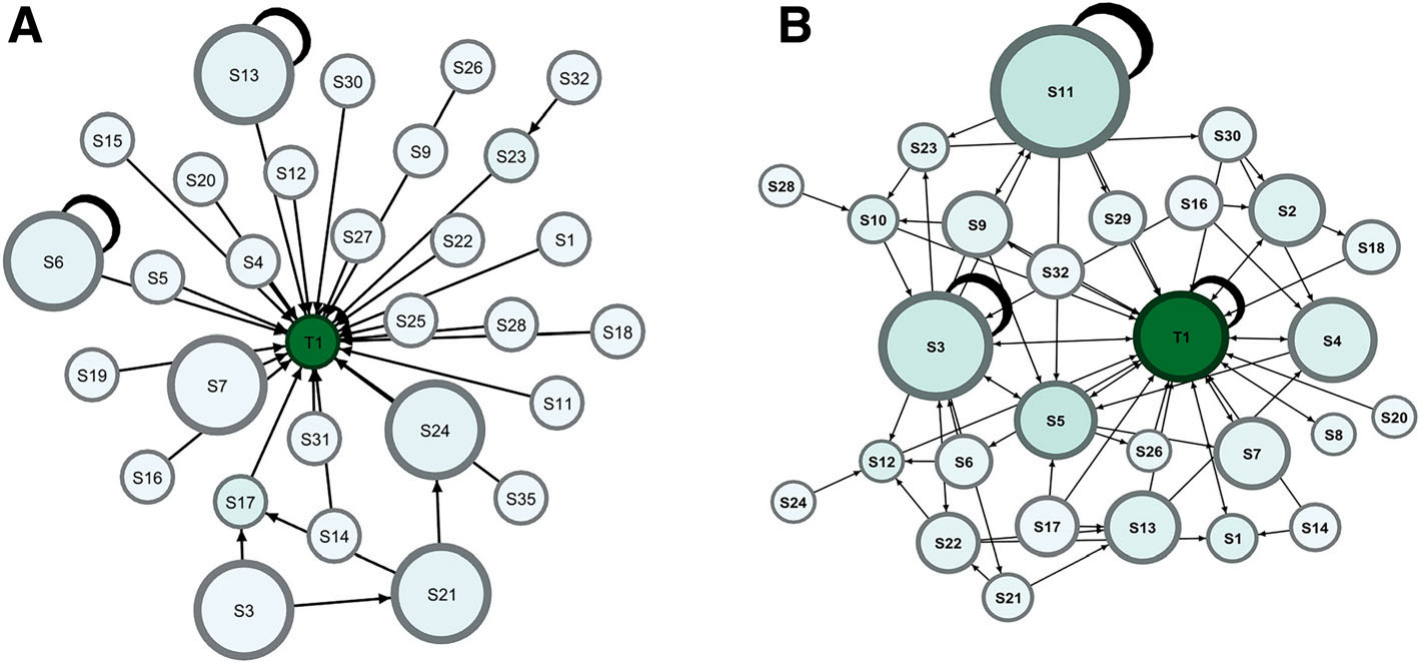
Graph (**a**) shows an instructor-centered discussion versus a participatory discussion in (**b**). Graph (**a**) shows an instructor-centered discussion where most interactions are directed towards the instructor. Graph (**b**) shows a more participatory discussion with numerous interactions among students. Each node (circle) corresponds to a participant, each edge (arrow) corresponds to an interaction, the arrowheads represent the direction of the interaction, the size of each node is relative to its degree centrality, color intensity represents betweenness centrality, and the thickness of edges represents the frequency of interactions

The course included 34 unique discussion threads, of which we demonstrate two. Figure 5a shows a typical instructor-centered discussion where most interactions are directed towards the instructor, and there are few interactions among students. On the other hand, Fig. 5b shows a more vibrant participatory discussion (student-centered) with numerous interactions among students. Several students’ nodes had a dark green color, which is an indication of importance (centrality) in passing the information throughout the discussion thread. The student-centered discussion threads were started by students and were more participatory.

#### At the individual level (ego network)

**Fig. 6:**
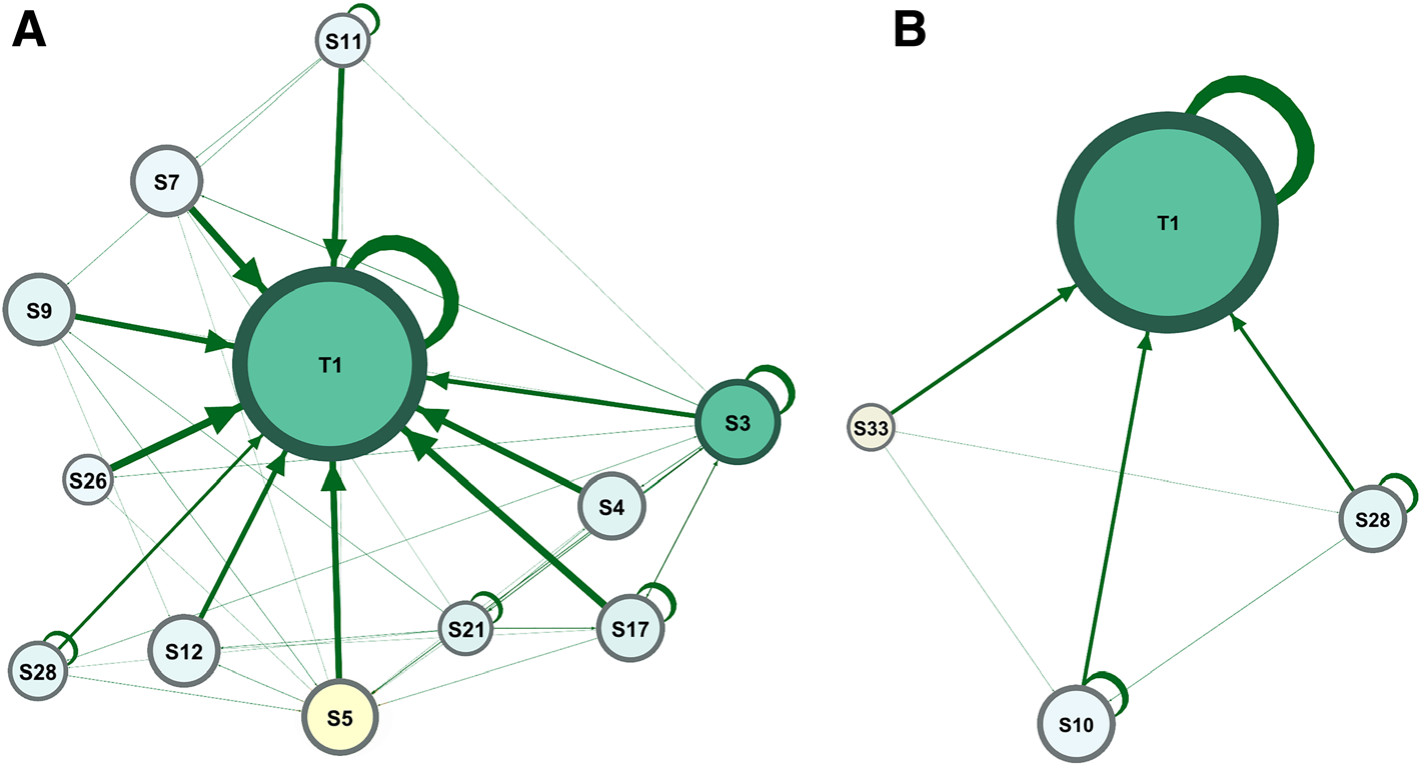
A graph showing the ego network of student S5 on the left (**a**) and S33 on the right (**b**). To the left, graph (**a**) shows the ego network of student S5 with a larger network of 11 neighbors and consequently more influence, compared to the small ego network of S33 on the right (**b**). Each node (circle) corresponds to a participant, each edge (arrow) corresponds to an interaction, the arrowheads represent the direction of the interaction, the size of each node is relative to its degree centrality, color intensity represents betweenness centrality, and the thickness of edges represents the frequency of interactions

Using SNA to profile a student can help understand the student’s social capital, personal ego network, and sphere of influence. The graphs in Fig. 6 show two students and the peers they interact with. One student (S5) is well-connected with a broad network of influence of 11 neighbors, while another (S33) has a network of only three neighbors, a small network of information exchange, and weak influence.

### Network properties

In this study, the course network size was 35 nodes (students) and 1251 edges (interactions). Students participated in 34 discussion threads, graph density was 0.14, the average degree was 69.5, and average clustering coefficient was 0.213. The low graph density is an indication of a low number of interactions among students, favoring the course instructor during the course. A full summary of the network characteristics is presented in Table 1.

**Table 1:** Shows a summary of the user parameters. (For variable definitions see section on Review of Social Network Analysis: Network analysis)

Parameter	Number	Minimum	Maximum	Mean	SD
Quantity of participation					
In-degree	35	0	29	7.2	5.48
Out-degree	35	4	78	34.54	13.19
Degree	35	4	107	41.74	18.03
Role in information relay					
Betweenness centrality^a^	35	0.00	0.39	0.02	0.07
Closeness centrality ^a^	35	0.36	0.76	0.44	0.10
Information centrality	35	0.85	3.90	2.66	0.70
Connectedness					
Eigenvector centrality ^a^	35	0.00	0.03	0.01	0.01
Eccentricity	35	2.00	4.0	3.57	0.61
Clustering ^a^	35	0.00	0.42	0.22	0.10

^a^ Scores are normalized so that 0 is the least and one is the highest

#### Research question 2

Which network parameters correlate best with students’ performance?

We investigated how much each student’s participation, interactions, and network parameters were correlated with the student’s academic performance. Two methods were used: Kendall’s Tau-b correlation between the SNA parameters and students’ grades, and automatic linear regression to see how much social network parameters could predict final grades or account for grade variance.

We found that parameters corresponding to the quantity of interactions (degree and out-degree) did not significantly correlate with student grade except for in-degree centrality, which was moderately significantly correlated (*τb* (33) = 0.32, *p* = 0.01), indicating that participating in discussions mattered when a participant created a contribution that stimulated peers to respond. All centrality scores measuring the role in information relay were positively correlated with final performance as well as Eigen centrality (*τb* (33) = 0.45, *p* < 0.001); Eigenvector centrality was also positively correlated with clinical grades (*τb* (33) = 0.38, p < 0.001), and MCQ grades (*τb* (33) = 0.37, p < 0.001). Full details of correlation are presented in Table 2.

**Table 2:** Correlation between centrality measures and students’ performance

Parameter	MCQs		Clinical exam		Final	
Quantity of participation	*τb*	*p*	*τb*	*p*	*τb*	*p*
In-degree	0.15	0.24	0.17	0.16	0.32^**^	0.01
Out-degree	0.16	0.18	0.10	0.40	0.16	0.17
Degree	0.18	0.13	0.12	0.34	0.23	0.05
Information relay						
Betweenness centrality	0.28^*^	0.02	0.46^**^	0.00	0.46^**^	0.00
Closeness centrality	0.11	0.35	0.20	0.1	0.27^*^	0.03
Information centrality	0.17	0.14	0.23	0.06	0.36^**^	0.00
Connectedness						
Eigenvector centrality	0.37^**^	0.00	0.38^**^	0.00	0.45^**^	0.00
Eccentricity	−0.10	0.49	−0.23	0.11	−0.29^*^	0.04
Clustering	0.12	0.33	0.05	0.7	0.23	0.06
Previous course	0.52^**^	0.00	0.35^**^	0.01	0.53	0.00

^*^Significant at the level of 0.05, ^**^Significant at the level of 0.01

#### Research question 3

How can student’s position, interactions, and relations in a network be used to predict his or her final performance?

Automatic linear regression (ALM) was used to test if SNA parameters can be used to predict the final grade and to what extent variance of grade can be explained by students’ participation. The adjusted R square (model accuracy) was 41.6%. The most important predictors were information centrality (26.71%) and Eigenvector centrality (21.27%). Predictor importance is a method by which SPSS characterizes the importance of each predictor and refers to the residual sum of squares if the predictor was excluded from the model. The values are normalized so that the sum is 100%. The accuracy of predicting clinical results was 51.3%, and the important predictors were Eigenvector centrality 40.85%, clustering 24.25% and betweenness centrality 23.10%. The model accuracy for predicting MCQ grades was 21.3%, the important predictors were Eigenvector centrality (39.62%) and information centrality (17.28%). More details are in Table 3.

**Table 3:** Predicting performance using SNA predictors by the end of course

3.1 Final Grade (Model accuracy 41.6%)	
Parameter	Importance %
Information centrality	26.71
Eigenvector centrality	21.27
Closeness centrality	12.69
Eccentricity	11.67
Out-degree	9.1
In-degree	6.62
Betweenness centrality	6.48
Clustering	5.45
3.2 Clinical results (Model accuracy 51.9%)	
Parameter	Importance %
Eigenvector centrality	40.85
Clustering	24.25
Betweenness centrality	23.10
Closeness centrality	3.41
Eccentricity	3.07
Out-degree	2.82
Information centrality	1.63
In-degree	0.87
3.3 MCQ (Model accuracy 21.3%)	
Parameter	Importance
Eigenvector centrality	39.62
Information centrality	17.28
In-degree	12.29
Out-degree	10.22
Closeness centrality	7.57
Clustering	7.18
Betweenness centrality	5.84

#### Early participation (midterm)

Interestingly, when centrality measures were calculated for the midterm, early participation was found to be more predictive of performance than it was with the whole course data: ALM accuracy for predicting midterm results was 71.6%, where out-degree, in-degree centrality, and Eigenvector centrality were the important predictors. For predicting final results, the model’s accuracy was 70%; the important predictors were information centrality, out-degree, in-degree centrality and Eigenvector centrality. Full details are presented in Table 4.

**Table 4:** Predicting performance using SNA predictors that were calculated at midterm

4.1 Midterm results (Model accuracy 71.6%)	
Parameter	Importance %
Out-degree	34.29
In-degree	33.54
Eigenvector centrality	14.17
Clustering	13.17
Closeness centrality	2.48
Betweenness centrality	1.8
Information centrality	0.55
4.2 Final Grade (Model accuracy 70%)	
Parameter	Importance %
Information centrality	23.87
Eigenvector centrality	17.65
In-degree	15.35
Out-degree	14.55
Closeness centrality	13.3
Betweenness centrality	9.73
Clustering	5.55

#### Can social network analysis contribute to predicting underachievers (at-risk)?

We included SNA predictors (Table 4.2) identified by ALM in the previous step as most significant **(**information centrality, Eigenvector centrality, in-degree, out-degree and closeness centrality) in addition to age, and previous performance in a stepwise backward binary logistic regression (BLR), to check if using early SNA indicators can contribute to predictability of low achievers. Full details are presented in Table 5.

**Table 5:** Cross-tabulation of predicted and low achievers, bold numbers denote correctly classified

Observed	Predicted	
	At-risk	Safe
At-risk	**8 (72.7%)**	3
Safe	2	**22 (91.7%)**

Using BLR, we were able to successfully classify 85.7% of students (91.7% of high achievers, 72.7% of underachievers) A chi-square test of independence was performed to examine the relation between actual and predicted at-risk students (chi-square = 15.3, *p* < .001, df = 1) (Cox & Snell R Square = 0.52, Nagelkerke R Square = 0.73, Hosmer and Lemeshow Test = 0.66). Both in-degree centrality (*P* = 0.02) and previous grade (*P* = 0.01) were statistically significant predictors.

## Discussion

Due to the novelty of social network analysis as a field, education-oriented SNA research has been very limited, and it has been mostly exploratory by nature. This has prompted for more exploration in varying disciplines using different methods [23, 45]. While most previous research concentrated on engineering or business education, this study is one of the first to study an online medical course using social network analysis [18, 19, 26, 32, 33, 45].

Using SNA visual analytics for analyzing learning can offer valuable insights on three levels: the course level, the discussion level, and the individual student level. On the course level, the mapped interactions identified gaps and pitfalls in the collaborative learning process, such as a dominating teacher, a relatively non-participatory network, and few interactions among students. This information offers opportunities for meaningful interventions to improve the status of collaborative learning in this course. Interventions can aim to raise awareness of the importance of participation [40], use relevant and flexible collaboration scripts, and train students to develop better social skills and communication practices [7, 33, 35, 39, 41, 42]. Teachers can help by scaffolding, supporting inclusive and supportive environments, stimulating interactive dialogues, and offering authentic problems that motivate argumentations and debate [9, 17, 35, 39, 42]. The course can be re-visualized using SNA to assess how effective the intervention was. Research has shown that teacher intervention might be necessary for certain small group situations, such as with dominating students or dysfunctional communications [7, 35]. On the thread level, monitoring individual discussions by means of SNA can help inform instructors about when to intervene: in the example we demonstrated, the non-participatory discussion was a candidate for such intervention.

On the individual level, the social capital, ego network, and the sphere of influence are of particular importance for students’ learning. Research has shown that students’ social capital is correlated with better academic performance [5, 41, 46, 47]. Using SNA to map the social profile of students can help identify an isolated student such as the inactive student in Fig. 6. Helping an isolated student improve his social skills might help promote his achievement [7, 37, 38].

The timeline of events (Additional file 1: Video S1) showed that the development of the online community was initiated by the instructor, students reacted enthusiastically along the course, and stopped around exam times. What is interesting was the instrumental role of the teacher presence that kept students engaged most of the course time, in contrast to the irregular and spiky patterns reported in non-moderated environments [14, 15, 60]. The sustained engagement during the course might have been the best advantage of having a moderator [14, 15, 20].

Analysis of network parameters can broaden our understanding of the various centrality measures in education and how they correlate with performance [13, 22, 37, 45]. Among centrality measures that reflect the quantity of participation, degree and out-degree centrality measures showed no correlation to performance, but in-degree centrality was significantly and moderately correlated with grades, as were centrality measures reflecting a role in information relay (betweenness, closeness, and information centrality). In-degree was also the only significant predictor of underachievement using logistic regression after controlling for age and previous performance. This is an indication that what mattered was how students were able to establish their cognitive presence as judged by their peers to be worth discussing [14, 15]. According to Chi et al. [61], interactions may be considered beneficial only when they generate knowledge that is beyond the presented learning materials and beyond other peer’s contributions, which deserves peers’ replies and discussion. Results also showed that the parameters reflecting group cohesion (high clustering coefficient and low eccentricity) were positively correlated with better performance.

Eigenvector centrality—a measure for the strength of neighbors (ego network) and social capital—were positively and significantly correlated with student final grades. MCQ and clinical performance emphasized the significance of personal networks and were in agreement with previous studies that underscored the value of social networks to academic performance [5, 41, 46, 47]. Regarding the prediction of the final grade, information and Eigenvector centralities were the most important predictors of final grade; MCQ and Eigenvector centrality accounted for 40% of clinical result predictability. Interestingly, early participation was more predictive of performance, with a model accuracy of 70%. It was apparent from the regression results that the weight of centrality measures varied according to the time it was measured.

Results from correlation coefficients and linear and logistic regressions indicate that a student’s position and role in information relay in online case discussions, combined with the strength of that student’s network (social capital), can be used as indicators of performance especially in relevant settings [37, 41, 46, 47]. This finding highlights a rarely studied potential of SNA parameters as early indicators of performance. The incorporation of SNA parameters in multi-dimensional learning analytics models can improve the predictive power of the models designed to identify students at risk of under-achievement in courses that makes use of online collaborative learning [45, 49].

These results are in accordance with similar studies in the field, although predictors vary from a study to another depending on the context and course design [37, 45, 48]. Nonetheless, measures of social capital showed agreement between our results and others [5, 41, 46, 47]. We concur with the previous studies in their belief that context and design play a major role. We believe that the significance of each indicator should be related to the framework of the time it was recorded, the setting of the online collaborative learning, and the social structure of the course (which is best understood by means of SNA). In this study, out-degree centrality was an early indicator of good performance. As the course advanced, in-degree centrality was more significant, as it represented an indirect vote of the quality of the contribution that garnered peers’ responses. In a course where the instructor was prominent (as demonstrated in the visual analytics), a prominent student was likely to be a successful one. The optimum use of SNA in evaluating online collaborative learning should not separate centrality measures from visual analytics, but rather combine them to better understand the context and interpret the inferences of each indicator.

The strength of SNA as a tool lies in the breadth of information it offers, which is relatively quick to produce and easy to interpret. Their speed and breadth of automatic analysis options stand in contrast to traditional content analysis methods, which require lengthy coding and manual analysis that make it impractical to implement it beyond research settings [62].

These results have many implications; firstly, for LMS designers to create ways that incorporate SNA in their systems so that teachers can monitor the social aspect of their courses; secondly, for administrators to harness the ever-growing field of learning analytics; thirdly, for teachers to seek training on new ways to monitor their work and that of their students to improve the collaborative work they offer; and lastly, for students who might benefit from SNA based intervention adaptive scaffolding.

In this research, we have shown ways to understand the dynamics of interactions in a clinical course in a medical college. This study is limited to a single course, which narrows the generalizability of the results. However, a broader understanding of complex collaborative environments requires studying the phenomena in a variety of contexts. Another limitation of this study is that we used SQL queries to extract interaction data from the database. This method was used due to two reasons. First, the LMS lacked built-in methods to extract the information at a sufficient detail. Second, SNA is still only a research endeavor, which has not found wide-scale adoption on the commercial level. Our study is a step into a new and growing field, which can only grow by extending research to new and unexplored areas.

## Conclusions

By using social network analysis, we were able to analyze a large number of interactions and discussions, gain an overall insight of the course social structure, and track knowledge flow and transfer. Mapping the networks of *information giving* and *information receiving* uncovered how information flows in the course, and identified important mediators in each network. The information centrality plot clearly demonstrated the influential actors in the network. When augmented by the calculated network parameters, it offered a precise view of the whole course network, each user’s position, level of connectedness, and participation. We were also able to estimate how much these interactions explained variance in learners’ grades, and what can improved in the course design.

Such insights are not possible using traditional methods, which only count hits or replies but ignore the importance of structure, relations, and interactions. This study demonstrates how SNA can expose invisible sides of online collaborative learning and how much social interaction affects learning.

Because this study is based on a case study of a medical education course, its generalizability is to be tested in terms of results and methodology. Future research should also focus on harnessing the power of SNA to improve learning through, for example, monitoring and intervention, early prediction of outliers, and improving social skills.

## Additional file

Additional file 1: Video S1. (MP4 7830 kb)
